# The oral microbiota and gestational diabetes mellitus

**DOI:** 10.3389/fcdhc.2023.1120920

**Published:** 2023-03-02

**Authors:** Jôice Dias Corrêa, Giovanna Araújo Faria, Leticia Ladeia Fernandes

**Affiliations:** Dentistry Department, Pontifical Catholic University of Minas Gerais, Belo Horizonte, Brazil

**Keywords:** oral microbiota, gestational diabetes, inflammation, periodontitis, pregnancy

## Abstract

Gestational diabetes mellitus (GDM) is one of the most frequent endocrine conditions during pregnancy. GDM is linked to adverse pregnancy outcomes and has implications for maternal health. Studies have demonstrated the link between pathogenic periodontal bacteria, glycemic control, and the risk of diabetes. The objective of the current study is to perform a mini-review of the available literature on the potential changes in the oral microbiota of women with GDM. The review was conducted by two independent reviewers (LLF and JDC). Indexed electronic databases (PubMed/Medline, Cochrane Library, Web of Science, and Scopus) were searched, including articles published in English and Portuguese. A manual search was also performed to identify related articles. The oral microbial community of pregnant women with GDM is unique from that of healthy pregnant women. The majority of the alterations found in the oral microbiota of women with GDM point to a pro-inflammatory environment with high levels of bacteria associated with periodontitis (*Prevotella, Treponema*, anaerobic bacteria) and a depletion of bacteria associated with periodontal health maintenance (Firmicutes, *Streptococcus, Leptotrichia).* More well-designed studies differentiating between pregnant women with good oral health and those with periodontitis are needed to ascertain which differences are due to GDM or periodontitis.

## 1 Introduction

As a unique physiological state, pregnancy causes temporary adjustments in a woman’s body composition, hormone levels, metabolism, and immune system. One important change is increased insulin resistance, due to the changes in placental hormone secretion that result in a higher demand for insulin secretion. However, some pregnant women have a reduced ability to produce more insulin, which can cause blood sugar levels to rise and, ultimately, result in GDM (1, 2). Obesity, a history of GDM, maternal age, polycystic ovary syndrome, multiple pregnancies, genetic factors, cigarette smoking, and a family history of diabetes are all risk factors for developing GDM (3). The gold standard for detecting GDM is an oral glucose tolerance test performed between 24 and 28 gestational weeks (3). The International Association of Diabetes and Pregnancy Study Groups (2010) recommended glucose thresholds of ≥180 mg/dl after one hour of oral glucose tolerance tests (3).

GDM is the most common pregnancy endocrine disorder, and its frequency varies greatly around the world, ranging from 1% to more than 30% (3). It is related to high−risk obstetric complications such as preeclampsia, miscarriage, premature delivery, fetal dysplasia, shoulder dystocia, or birth injury, and fetal growth restriction with clinical neonatal hypoglycemia (3). In neonates, GDM can lead to respiratory distress syndrome, fetal macrosomia, obesity, and Type II diabetes (3–6).

High levels of tumor necrosis factor (TNF), C-reactive protein (CRP), and interleukin 6 (IL-6) point to the involvement of infection and inflammation in the etiology of GDM. GDM has been linked to microbial changes in the maternal microbiome at various body locations (6). The majority of the studies on GDM and human microbiota have focused on the gut microbiota, and research has demonstrated that intestinal microbiota may cause insulin resistance by affecting the inflammatory response (6, 7). Other studies have shown that there is an increase in oral microbial load during pregnancy, which leads to oral microbiota dysbiosis (8). In this way, dysbiosis in this community often results in the development of dental diseases such as periodontitis. Like GDM, periodontal disease has been linked to preterm birth and low birth weight (9). Furthermore, there is evidence linking pathogenic periodontal bacteria with glycemic control and the risk of diabetes (10, 11). In addition, some studies have shown an association between GDM and periodontitis (9).

The present study aims to perform a review of the available literature on the possible changes in the oral microbiota of pregnant women with GDM and their associations with GDM pathology.

## 2 Material and methods

The review was conducted by two independent reviewers (JDC and GAF). Indexed electronic databases (PubMed/Medline, Cochrane Library, Web of Science, and Scopus) were searched, including articles published in English and Portuguese. The terms used in the search were “gestational diabetes” and “oral microbiota.” A manual search was also performed to identify related articles.

The inclusion criteria were epidemiological studies (cross-sectional, case-control, cohort, and clinical trials) analyzing the oral microbiota in women with GDM, including the presence of a control group of pregnant women without GDM. A total of 32 studies that evaluated intestinal microbiota, newborn microbiota, treatments, and literature reviews were excluded.

## 3 Differences in the oral microbiota in GDM

The literature search resulted in nine studies that looked at the oral microbiota of pregnant women with GDM. Data from each study and their main results are summarized in **Table 1**. The first study dates back to 2008, but there has been a large gap in research interest in the area, with new publications appearing only 10 years later, in 2018. It was challenging to compare the trials because different clinical and laboratory protocols were used. Six studies utilized more recent molecular methods (16S rDNA sequencing) to identify oral microbial communities, two used PCR to identify specific bacteria, and one identified bacteria by culturing the oral samples.

**Table 1:** Summary of oral microbiota in GDM studies.

Study	Groups	Sample source	Microbial detection methods	Oral examination	Bacteria depleted in GDM	Bacteria increased in GDM
12	22 GDM and 240 GDM-	Subgingival plaque	PCR	Yes	No difference	No difference
4	149 GDM and 197 GDM-	Saliva	16S rDNA sequencing	No	Firmicutes and Leptotricia	Proteobacteria and Neisseria
13	59 GDM and 59 GDM-	Supragingival and subgingival plaque	Culturing	yes	Oral streptococci and lactobacilli	Oral anaerobic bacteria, tuberculosis bacilli, Black-pigmented bacteria, and Capnocytophaga, actinomycetes
1	26 GDM and 42 GDM-	Saliva	16S rDNA sequencing	No	No difference	No difference
14	26 GMD and 26 GDM-	Subgingival plaque	PCR	Yes	Not reported	P. gingivalis and Prevotella intermedia
2	50 GDM and 161 GDM-	Saliva	16S rDNA sequencing	No	Actinobacillus paraheamolyticus, Neisseria, Streptococcus	Prevotella, Veillonellaceae, Bacteriodales, and Treponema
7	30 GDM and 31 GDM-	Saliva	16S rDNA sequencing	No	Leptotrichia and Fusobacterium	Selenomonas and Bifidobacterium
5	44 GDM and 67 GMD-	Saliva and plaque	16S rDNA sequencing	Yes	Firmicutes, Streptococcus and Veillonella, Selenomonas, and Leptotrichia	Proteobacteria, Leptotrichiaceae, Lautropia, Neisseria, Neisseriales, andCapnocitophaga
6	14 GDM and 55 GMD-	Saliva and plaque	16S rDNA sequencing	Yes	Not reported	Bacteroides eggerthii, Ruminococcaceae, and Enterobacteriaceae

The majority of studies have indicated significant differences in the oral microbiota of women with GDM compared to women with healthy pregnancies. Only two studies did not report differences (1, 12). There was agreement among the studies in reporting an increase of some oral bacteria in women with GDM, including *Proteobacteria* and *Neisseria* (4, 5), *Prevotella* (2, 14), and *Capnocytophaga* (5, 13). The same results were also found for bacteria depleted in GDM: *Streptococcus* (2, 5, 13), *Firmicutes* (4, 5), and *Leptotricia* (4, 5, 7). On the other hand, some studies have shown divergent results in the proportion of bacteria found in different groups. While *Selenomonas* was depleted in the GDM group in the study by Li et al. (5), it was found at increased levels in healthy pregnancies compared to GDM in the study by Xu et al. (7). Similarly, *Neisseria* was found to be depleted in GDM compared to healthy women in the study by Xu et al. (7).

## 4 Discussion

The data gathered here indicate that the oral microbiota of women with GDM is different from that of women with healthy pregnancies. In this way, numerous studies have shown a link between GDM and periodontitis. This could play a role in the insulin resistance seen in GDM women. According to reports, the incidence of GDM is higher in people with periodontitis (9), although it is unclear whether changes in the microbiome are what connect these two diseases. The results of this review indicate that the majority of the alterations found in the oral microbiota of women with GDM favor an inflammatory environment with high levels of bacteria associated with periodontitis (*Prevotella, Treponema*, anaerobic bacteria) and a depletion of bacteria associated with the maintenance of periodontal health (Firmicutes, *Streptococcus, Leptotrichia*) (15, 16).

Both pregnancy and diabetes are known to have effects on oral microbiota (17, 18). Previous studies suggested that changes occur in the oral microbiota during pregnancy due to modifications to hormone (estrogen, progesterone, and gonadotropin) levels (8). In fact, a meta-analysis recently confirmed an increase in the total salivary bacteria in pregnant women (8) and that hormonal changes during pregnancy increase the development of certain Gram-negative anaerobic bacteria in the mouth (18).

Similarly, periodontal bacteria, particularly anaerobic bacteria, benefit greatly from hyperglycemia (19). The high availability of glucose may support higher levels of saccharolytic bacteria, promoting the proliferation of fermenting species and creating a selective environmental pressure on glucose availability because the amounts of glucose in the gingival crevicular fluid and serum are similar (20). According to a study on mice, the oral bacterial makeup clearly changed as the animals developed diabetes, with higher amounts of Proteobacteria ( 21), which is consistent with the results shown here. In a previous study, they also reported that suppressing Interleukin-17 caused the bacterial population of diabetic mice to resemble more closely that of normoglycemic mice (21). In addition, diabetes increases the levels of glucose, advanced glycation end-products, and reactive oxygen species in periodontal tissues. In gingival fibroblasts, advanced glycation end-products have been shown to stimulate the production of inflammatory cytokines such as tumor necrosis factor, interleukin-1beta, interleukin-17, interleukin-23, and interleukin-6 (22).

This increase in systemic inflammation and periodontal tissues may induce an imbalance in the oral microbiota because, as previously demonstrated, an inflammatory environment favors the growth of pathogenic bacteria associated with periodontitis (23). Thus, there is a link between insulin resistance and the development of periodontal disease during pregnancy (24). The proposed mechanism is due to the systemic dissemination of inflammatory mediators derived from periodontal disease. Growing evidence suggests that periodontal organisms and their virulence factors cause increased systemic inflammation, including higher levels of CRP, TNF-alpha, and IL-6 (25). Due to their antagonistic action against insulin, these inflammatory mediators have been shown to have a significant impact on glucose metabolism. Continuously elevated levels of IL-6 and TNF-alpha can disrupt carbohydrate metabolism, resulting in glucose intolerance and gestational diabetes mellitus (9).

**Figure 1:**
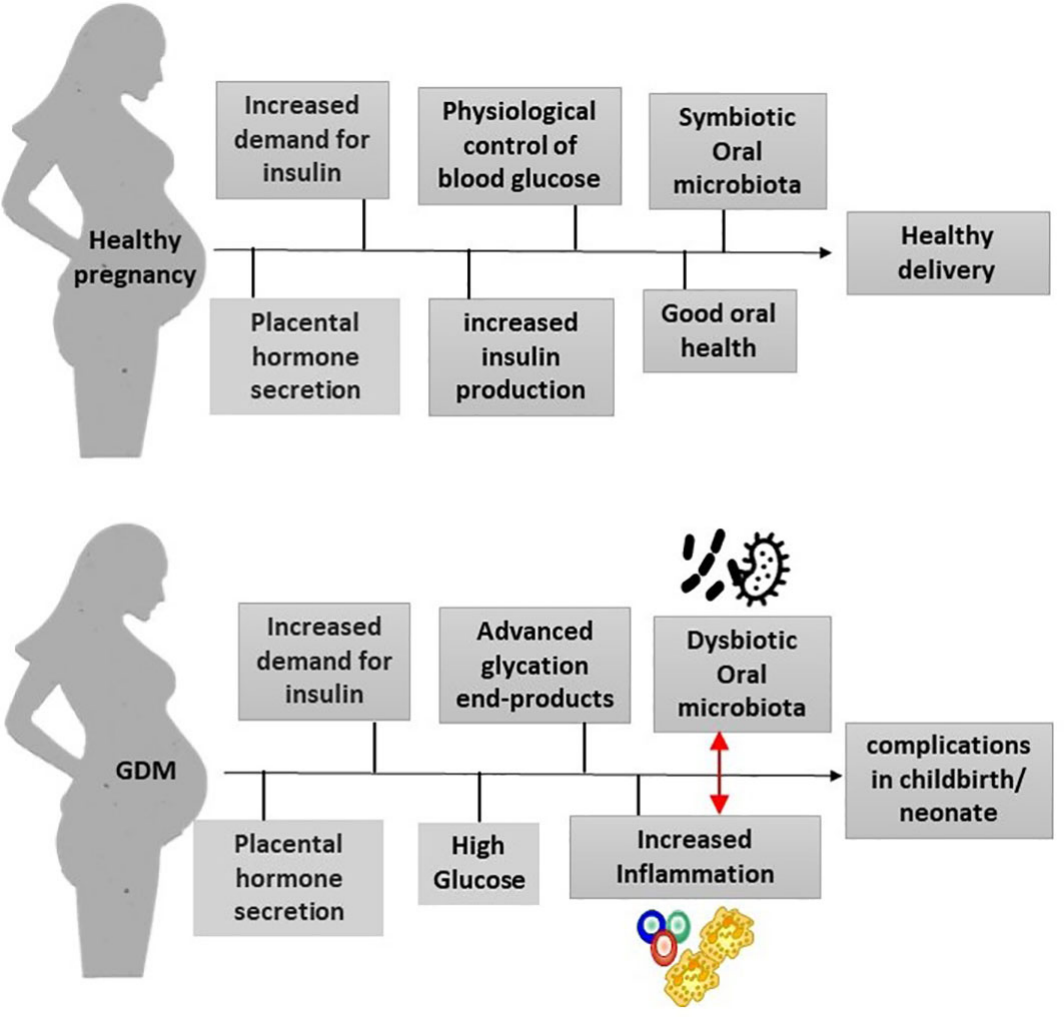
Mechanisms that link GDM and oral microbiota. In healthy pregnancies, there is an increased need for glucose, leading to an increase in insulin production. The pregnant woman’s body manages to control blood glucose levels, and the pregnancy is healthy. Without alterations in the oral microbiota and with good oral health, there will be no pregnancy complications. GDM raises glucose and advanced glycation end-product levels in periodontal tissues. This causes inflammation, which impacts the oral microbiota, leading to dysbiosis. In turn, dysbiosis increases inflammation, which also affects glucose control.

Thus, what seems to occur is a repetitive cycle that perpetuates the changes seen in GDM, in which increased levels of glucose and advanced glycation end-products lead to increased inflammation, which induces dysbiosis of the oral microbiota. Dysbiosis, in turn, leads to more periodontal inflammation, which has a systemic impact and consequently worsens diabetes (**Figure 1**).

However, because GDM is multifactorial, it is important to analyze other risk factors in research. Maternal age and BMI can be confounding variables in GDM studies because both are considered risk factors for the development of this condition. According to some of the studies reviewed, women with GDM had a higher mean age than pregnant women without GDM (1, 6), while others found no differences (2, 7, 13). Unfortunately, not all studies assessed BMI (4). Other findings indicated that women with GDM had a higher BMI (1, 12), while others found no differences in BMI between women with GDM and healthy pregnant women (2, 5–7, 13).

Maternal oral health is closely associated with children’s oral health, including maternal relatedness and the vertical transmission of oral pathogens from mothers to infants. Studies have shown that when a mother has GDM, the microbiota of her newborns is altered, which is very similar to the change that occurs in the oral microbiota of mothers. Thus, it is hypothesized that the effects of GDM on the maternal microbiota can be vertically transmitted to the fetus during pregnancy (26, 27). These differences could have a high impact on children’s lives, as research has shown a link between microbial dysbiosis during childhood and a variety of diseases (28).

One limitation of this review is that the results of the studies differ and, in some cases, contradict each other. The difficulty in comparing results may be attributed to the complexity of GDM as a multifactorial disease, the genetic differences between study populations, differences in the methods used to identify bacteria, and the fact that some studies did not evaluate oral health prior to sampling. The sites collected in the studies differ in subgingival, supragingival, tongue, and saliva. The Human Microbiome Project has already demonstrated that there is a significant difference between these intraoral sites, which may affect the bacteria found in the studies (29). Another limitation is that some studies use culture-based bacterial identification methods, while others only analyze specific bacteria. Therefore, the results of these investigations are limited in comparison to studies using bacterial DNA sequencing methods, which allow us to examine the general picture of microorganisms with a greater understanding of the changes that occur during GDM.

The topic in this review is relatively new, with the majority of studies conducted since 2018, and interest in the possible link between oral microbiota and GDM has grown in recent years. Unfortunately, most works have not evaluated the microbiota of periodontitis or healthy periodontium separately. More well-designed studies separating pregnant women with good oral health from those with periodontitis are needed to ascertain which differences are due to GDM or periodontal conditions, as it is unclear whether the identified changes are primarily caused by periodontal inflammation or by hyperglycemia.

Therefore, understanding the risk factors associated with GDM is critical to reducing the complications associated with this condition.

## 5 Conclusion

We can conclude from the results observed in this review that the oral microbiota of pregnant women with GDM is different from that of healthy pregnant women, and this discrepancy usually favors pathogenic bacteria, indicating a dysbiosis of the oral microbiota. Pregnant women with GDM should be carefully monitored by a multidisciplinary team led by a periodontist since high levels of periodontal pathogens during pregnancy have been linked to an increased risk of preterm delivery.

## Author contributions

JC: conceptualized the project, assisted with article search and wrote the text. GF and LF: conducted the search for articles, summarized the findings and assembled the results. All authors contributed to the article and approved the submitted version.
